# Breed and Season: Key Determinants of Efficiency in Large-Scale Commercial *In Vitro* Sheep Embryo Production

**DOI:** 10.3390/ani15223354

**Published:** 2025-11-20

**Authors:** Yubing Wang, Ke Li, Jia Hao, Dayong Chen, Lei Cheng, Huijie He, Riga Wu, Yingjie Wu, Jianhui Tian, Guangyin Xi

**Affiliations:** 1 Laboratory of Animal Genetics, Breeding and Reproduction of the Ministry of Agriculture and Rural Affairs, Frontiers Science Center for Molecular Design Breeding (MOE), College of Animal Science and Technology, China Agricultural University, Beijing 100193, China; wangyubing911@163.com (Y.W.); like5726@outlook.com (K.L.); 17630751829@163.com (J.H.); wuyingjie@cau.edu.cn (Y.W.); 2 Key Laboratory of Sheep Genetic Breeding and Reproduction Technology, Ministry of Agriculture and Rural Affairs, Siziwang Banner, Ulanqab 011800, China; chendayong81@126.com (D.C.); cl344713520@163.com (L.C.); 15904749394@163.com (H.H.); wuriga000@126.com (R.W.)

**Keywords:** sheep breed, in vitro embryo production, oocyte, laparoscopic ovum pick up, seasonal reproduction, embryo transfer

## Abstract

The efficiency of the laparoscopic ovum pick-up and in vitro embryo production (LOPU-IVEP) system in sheep is governed by a complex interplay of genetic and environmental factors. This study comprehensively evaluated the effects of breed, season, and their interaction on oocyte retrieval, embryonic development, and pregnancy outcomes in a commercial setting. A 20-month longitudinal analysis of a large commercial cohort revealed that breed was the dominant factor determining oocyte quantity, whereas season exerted a stronger influence on developmental competence. Specifically, autumn and winter provided optimal conditions, resulting in significantly higher cleavage rate and blastocyst rate (*p* < 0.05). A significant breed-by-season interaction further identified synergistic combinations, such as Black-headed Suffolk in autumn or winter, which achieved superior embryo production outcomes. Importantly, correlation analysis revealed that oocyte quantity was not predictive of developmental potential or pregnancy success. These findings underscore the necessity of a precision management strategy that aligns LOPU schedules with breed-specific seasonal advantages to maximize annual embryo production efficiency.

## Simple Summary

Reproductive technologies like in vitro embryo production (IVEP) allow farmers to rapidly multiply the best genetics in their sheep flocks. A key step is collecting oocytes from valuable donor ewes using a technique called laparoscopic ovum pick-up (LOPU). However, the success of this process can vary. Our study investigated how the sheep’s breed and the season affect the entire production chain, from oocyte collection to the birth of a lamb via embryo transfer. We found that the best outcomes are achieved by matching specific breeds to their optimal season. The breeds demonstrated a clear functional division of labor: some were identified as superior oocyte donors, while others showed greater efficacy in supporting pregnancy. Crucially, we show that autumn and winter are the most productive seasons. By aligning breeding schedules with these breed-season combinations, sheep farmers can significantly improve the efficiency and success of their advanced breeding programs.

## 1. Introduction

According to data from the International Embryo Transfer Society (IETS), global IVEP in sheep increased by 62.3% between 2022 and 2023 [1]. This trend reflects a paradigm shift from traditional in vivo embryo production to in vitro embryo production (IVEP), driven by the latter’s capacity to enhance production efficiency, shorten reproductive cycles, and improve genetic selection precision. The IVEP process comprises several critical stages: in vitro oocyte maturation (IVM), in vitro fertilization (IVF), and in vitro embryo culture (IVC) to the blastocyst stage, followed by embryo transfer or cryopreservation.

Despite its advantages, the overall efficiency of the sheep IVEP system remains highly variable. Blastocyst rates in sheep IVEP typically range from 15% to 79% [2], influenced by complex factors including culture media [3,4], follicle size [4,5,6], donor reproductive status, semen quality [7,8,9], oocyte retrieval methods [10,11,12], and breeding season [12,13]. Notably, seasonal variations in embryo yields have been widely documented, with oocytes collected during breeding seasons exhibiting superior developmental competence compared to non-breeding periods [12,13,14]. Seasonal effects on fertilization, cleavage, and blastocyst rates in sheep IVEP are well-established [15,16,17,18], a phenomenon also observed in goats, buffalo, and cats [19,20,21,22,23,24,25,26,27,28]. Sheep, as seasonally polyestrous species, exhibit reproductive efficiency modulated by photoperiodic changes [18]. In the Northern Hemisphere, breeding seasons include transitional (June–September) and peak periods (September–December) [16]. The physiological mechanism is orchestrated by the pineal gland, which translates decreasing daylight into increased melatonin secretion. This neurohormone plays a critical role in regulating circadian rhythms and, crucially, stimulates the hypothalamic-pituitary-gonadal (HPG) axis by promoting the release of Gonadotropin-Releasing Hormone (GnRH). This cascade ultimately elevates circulating levels of Luteinizing Hormone (LH) and Follicle-Stimulating Hormone (FSH), thereby synchronizing ewes into a state of heightened reproductive activity and oocyte competence [29,30]. The increase in melatonin further stimulates the rise in GnRH levels, subsequently leading to elevated LH and FSH levels, which trigger cyclical reproductive changes [31,32].

However, a critical gap exists in the current understanding. The majority of studies investigating seasonal effects are based on oocytes sourced from abattoirs. This approach introduces significant confounding heterogeneity due to variable retrieval techniques, uncontrolled transportation conditions (time and temperature), and unstandardized follicle selection criteria [23]. Moreover, the absence of controlled follicular hormone induction in such models limits their representativeness for organized, live-animal commercial production systems. Most importantly, while season is a recognized factor, the interaction between season and genetic background (breed) in a large-scale commercial context remains largely unreported. Different breeds may possess distinct genetic adaptations that modulate their response to seasonal photoperiodic cues, yet this potential interaction is often overlooked.

Therefore, this study was designed to address this specific knowledge gap. We aimed to systematically investigate the individual and interactive effects of breed and season on the entire LOPU-IVEP pipeline within a large-scale commercial system. In addition, a systematic analysis was conducted on the embryo transfer pregnancy rates of different breeds in different seasons. By establishing a comprehensive correlation between these factors, this research provides the foundation for precision management strategies to optimize annual embryo production efficiency and enhance the economic returns of commercial sheep breeding.

## 2. Materials and Methods

### 2.1. Experimental Animals, Location, and Time

The ewes were fed in the Inner Mongolia Sino sheep Technology Co., Ltd., Wulanhua Town Siziwang Banner, Inner Mongolia, China (111:66E, 41:55N). All animals in the semi-intensive farms were kept indoors during the night and some part of the day and were moved to the pastures during some period of the day. The total mixed ration (TMR) provided contained approximately 14.5% crude protein and 2.51 Mcal of metabolizable energy (ME) per kg of dry matter, formulated with 55% corn silage, 20% alfalfa hay, and 25% concentrate (dry matter basis). Each donors had health and estrous cycling of an expected duration. All donor and recipient ewes were multiparous adults aged 2–4 years. Donor ewes were reused with a minimum 60-day recovery interval between LOPU sessions. Random assignment to seasonal groups prevented individual bias. The five sheep breeds utilized in this study were strategically selected to capture a wide spectrum of commercially relevant and physiologically distinct genetic profiles. This deliberate diversity was essential for rigorously investigating breed-season interactions. The East Friesian served as a benchmark for high oocyte yield and prolificacy. The Black-headed Suffolk was chosen for its exceptional maternal traits and oocyte developmental competence, while its White-headed counterpart provided a valuable genetic comparator within the Suffolk lineage. The Australian White represented a breed documented for heat tolerance, and the Black-headed Dorper contributed tropically adapted hair sheep genetics. This strategic selection created a comprehensive genetic gradient encompassing dairy versus meat production, temperate versus tropical adaptation, and high oocyte yield versus high developmental quality, thereby enabling a robust examination of how genetic background modulates seasonal responses in a LOPU-IVEP system [33].

### 2.2. Oocyte Collection

Oocytes were collected via laparoscopic ovum pick-up (LOPU) following a standardized superovulation protocol [10]. Donor ewes received an intravaginal progesterone-impregnated sponge (Sansheng, Ningbo, China) at a random stage of the estrous cycle, designated as Day 0. From Days 10 to 12, ewes were administered FSH (Sansheng, Ningbo, China) intramuscularly twice daily at 12 h intervals, with a total dose of 480 IU. Laparoscopic oocyte collection was performed 12 h after the sixth FSH injection, after which the sponge was removed [34]. Prior to the procedure, animals were fasted for 12 h in preparation for general anesthesia. Donors were anesthetized with 0.12 mL/kg of xylazine-based anesthetic (Su-Mian-Xin II; Institute of Military Veterinary, Changchun, China) and positioned in Trendelenburg position. Following ventral abdominal shaving and disinfection with 1% iodine solution (LIRCON, Dezhou, China), a 10 mm laparoscope (Olympus Winter & Ibe GmbH, Hamburg, Germany) was introduced through the ventral midline. After ovarian visualization, ovaries were exteriorized and stabilized with atraumatic grasping forceps. All follicles ≥ 2 mm in diameter were aspirated using a 20-G needle connected to a vacuum system maintained at 20–25 mmHg. The aspiration medium consisted of TCM-199 supplemented with 0.3% (*w*/*v*) bovine serum albumin (BSA, Sigma Chemicals Co., St. Louis, MO, USA), 100 IU/mL penicillin (Thermo Fisher Scientific, Shanghai, China), and 6 IU/mL heparin (Thermo Fisher Scientific, Shanghai, China), maintained at 38 °C. Oocytes were transported immediately after LOPU collection through a dedicated pass-through window connecting the surgical suite directly to the laboratory isolation area. To prevent post-operative adhesions, the ovarian surface was rinsed with warmed metronidazole-saline solution before instrument removal. All donors received a preventive dose of 20 mg/kg ampicillin sodium (Harbin Pharmaceutical Group Co., Ltd., Harbin, China) post-operatively [35].

### 2.3. Oocyte Evaluation

**Figure 1:**
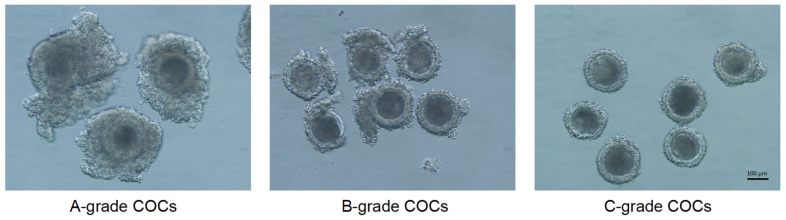
Representative images of different grade COCs (scale bar = 100 μm).

Oocytes retrieved via LOPU were morphologically graded under a stereomicroscope (SMZ-645; Nikon, Tokyo, Japan) following Wieczorek’s classification criteria [26]. The grading system evaluated COC integrity based on cumulus cell layers and cytoplasmic homogeneity in Figure 1:

Grade A: ≥3 compact cumulus cell layers with homogeneous cytoplasm.

Grade B: 2–3 partially expanded cumulus cell layers with homogeneous cytoplasm.

Grade C: 1 discontinuous cumulus cell layer with homogeneous cytoplasm.

COCs meeting minimum viability thresholds (≥1 intact cumulus cell layer with granular cytoplasm, Grades A–C) were selected for subsequent IVM procedures. This screening protocol ensured exclusion of denuded oocytes and those exhibiting cytoplasmic abnormalities (vacuolization or darkening).

### 2.4. In Vitro Maturation

The oocytes were washed three times in maturation medium, and incubated at 38.6 °C in a humidified environment containing 5% CO_2_ and 95% air for 24 h. The maturation medium consisted of TCM-199 (Gibco, Grand Island, NY, USA) supplemented with 10% fetal bovine serum (Gibco, Grand Island, NY, USA), 0.02 U/mL FSH (Sansheng, Ningbo, China), 0.02 IU/mL LH (Sansheng, Ningbo, China), 1 μg/mL 17β-estradiol (Sigma-Aldrich), 100 μM cysteamine (Sigma-Aldrich, St. Louis, MO, USA), and 1% penicillin-streptomycin (*v*/*v*) (Gibco, Grand Island, NY, USA). And then approximately 50 COCs were incubated in a 4-well culture dish (Thermo Scientific, Roskilde, Denmark) that contained 600 μL of the same maturation medium covered with 300 μL mineral oil (Sigma-Aldrich, St. Louis, MO, USA).

### 2.5. In Vitro Fertilization, and In Vitro Culture

Following IVM, the cumulus cells surrounding the oocytes were removed by repeated pipetting in synthetic oviductal fluid (SOF; Caisson Laboratories, Rexburg, ID, USA) containing 0.2% (*w*/*v*) hyaluronidase (Sigma-Aldrich, St. Louis, MO, USA). The denuded oocytes were washed twice and subsequently transferred into 100 µL droplets of pre-equilibrated IVF medium, which was SOF supplemented with 2% (*v*/*v*) oestrous sheep serum, 3 mg/mL BSA, 6 IU/mL heparin sodium, and 50 IU/mL gentamicin. The droplets were covered with 300 µL of mineral oil (Sigma-Aldrich, St. Louis, MO, USA).

Frozen semen from breed-specific rams was thawed in a 39 °C water bath for 1 min. Motile spermatozoa were selected using a swim-up procedure. Briefly, the thawed semen was diluted in pre-equilibrated IVF medium, and the motile sperm fraction was isolated by a 30 min swim-up incubation at 38.5 °C under 5% CO_2_ in humidified air. The upper layer, enriched with motile sperm, was collected and centrifuged at 200× *g* for 5 min. The resulting sperm pellet was resuspended in fresh IVF medium and adjusted to a final concentration of 2 × 10^6^ spermatozoa/mL. For fertilization, the prepared sperm suspension was introduced into the fertilization droplets containing the oocytes. Gametes were co-incubated for 20 h at 38.5 °C under 5% CO_2_ and maximum humidity.

Presumptive zygotes were washed three times in IVC medium and cultured in groups of 25–30 in 50 µL droplets of SOF supplemented with 1% (*v*/*v*) BME-essential amino acids, 1% (*v*/*v*) MEM-nonessential amino acids, 1 mM l-glutamine and 3 mg/mL BSA under 38.5 °C, 88% N_2_, 5% CO_2_ and 7% O_2_ [7]. Cleavage and blastocyst rates were recorded at 48 h and on day 6 post-IVF, respectively.

### 2.6. Embryo Transfer

Morphologically normal blastocysts at day 6–7 of development were selected for transfer. Embryo transfer was performed according to the standard procedures recommended in the International Embryo Technology Society (IETS) Manual, 4th Edition. All recipient ewes were of the Mongolia sheep breed. To synchronize the reproductive cycles of the recipient and donor ewes, both received intravaginal progesterone sponges (Sansheng, Ningbo, China) on the same day. Sponges were removed from the recipient ewes at the time of the fifth FSH injection in the donors, and each recipient was intramuscularly administered 330 IU PMSG (Pfizer, New York, NY, USA). All recipients were fasted for 24 h prior to surgery. Embryo transfer was performed via a laparoscopic approach [36,37]. The uterine horn was exteriorized, and a sterilized blunt-tipped needle was used to create a small perforation at its distal end. Either a single blastocyst or two blastocysts, depending on the pre-assigned experimental group, were deposited into the uterine lumen through this aperture using an embryo transfer catheter. Following the procedure, each ewe received an intramuscular injection of 2.5 mg progesterone (Sansheng, Ningbo, China) to support luteal function. Following the procedure, all instruments were removed and a preventative dose of 20 mg/kg ampicillin sodium (Harbin Pharmaceutical Group Co., Ltd., Harbin, China) was administered. Pregnancy status was determined by trans-abdominal ultrasonography 40 days after transfer, with conception confirmed by visualization of embryonic vesicles and heartbeat.

### 2.7. Statistical Analysis

All statistical analysis were performed using SPSS (version 22.0; IBM Corp., Armonk, NY, USA). Data are presented as mean ± standard deviation (SD), and a probability value of *p* < 0.05 was considered statistically significant.

Generalized linear models (GLM) with a Poisson distribution and log-link function were employed to analyze count data, including the number of total, available, grade A, grade B, and grade C COCs per ewe. The models included the fixed effects of breed, season, and their interaction. Proportion data (cleavage rate, blastocyst rate, and developmental efficiency) were arcsine square-root transformed and then analyzed via two-way ANOVA, considering breed, season, and their interaction. For pregnancy outcomes (single- and double-embryo transfer pregnancy rates), a binary logistic regression model was fitted with breed, season, and their interaction as categorical predictors. The presented chi-square values are Wald statistics testing the significance of fixed effects in the models.

For significant fixed effects, post hoc pairwise comparisons were conducted: Tukey’s HSD test was applied for ANOVA models, while analyses were performed on estimated marginal means with Sidak adjustment for GLMs (Poisson and logistic regression).

## 3. Results

### 3.1. The Significant Breed-Season Interaction Reveals Non-Uniform Responses in IVEP

The comprehensive analysis revealed distinct and independent roles of breed and season on the LOPU-IVEP pipeline (Table 1, Table 2 and Table 3). Breed was identified as the primary driver of oocyte recovery and quality, whereas season exerted a more pronounced influence on subsequent embryonic developmental competence and pregnancy outcomes. A significant breed-by-season interaction was detected for key developmental milestones, indicating that the performance of specific breeds varied across different seasons.

**Figure 2:**
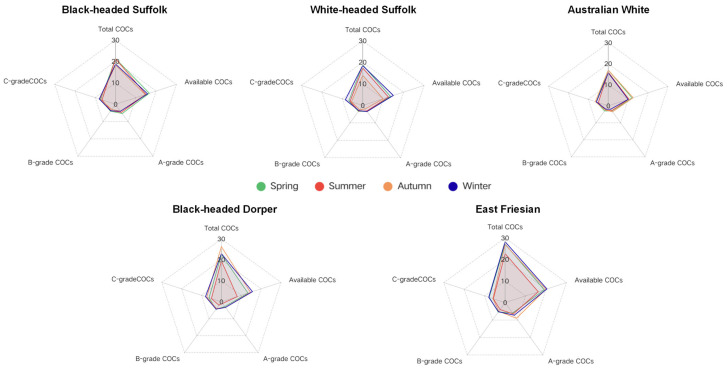
Visualization of breed × season interaction on COCs yield and quality. Radar charts depicting the seasonal profile of oocyte retrieval outcomes for different sheep breeds.

Regarding oocyte retrieval, breed had a highly significant (*p* < 0.001) impact on the number of total COCs, available COCs, and grade A COCs per ewe (Table 1 and Table S1). In contrast, season and the breed-season interaction showed no significant effects on any COCs quantity or quality parameters. The seasonal patterns of oocyte yield and quality across different breeds are visually summarized in Figure 2, which illustrates that East Friesian exhibited consistently superior LOPU efficiency, while Australian White performed significantly lower than other breeds.

**Figure 3:**
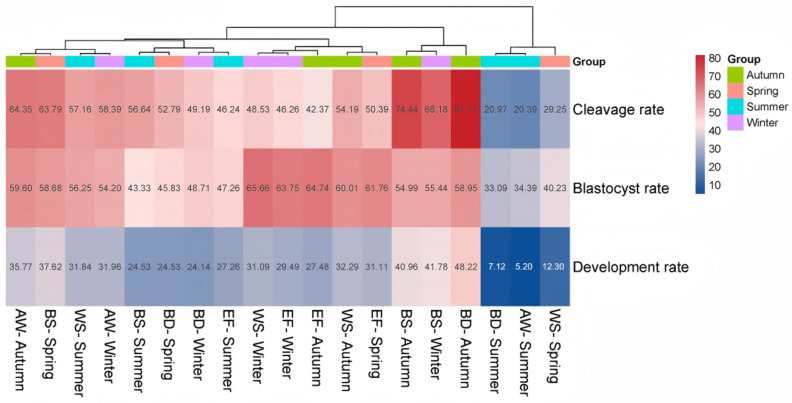
Cluster analysis of embryonic development efficiency across breed-season combinations. Heat map depicting the performance of different sheep breeds across seasons based on in vitro embryonic development criteria. BS, Black-headed Suffolk; WS, White-headed Suffolk; AW, Australian White; BD, Black-headed Dorper; EF, East Friesian.

For in vitro embryonic development, both breed and season significantly affected the cleavage rate (*p* < 0.001 for both). Season was also a strong determinant of development rate (*p* < 0.001). Crucially, a significant breed × season interaction was observed for both cleavage rate (*p* = 0.001) and developmental rate (*p* = 0.029), indicating that the developmental potential of oocytes from different breeds varied seasonally (Table 2 and Table S2). Cluster analysis (Figure 3) further identified Black-headed Suffolk in autumn and winter, and Black-headed Dorper in autumn as superior combinations for embryonic development. Conversely, the combinations of Black-headed Dorper in winter, Australian White in summer, and White-headed Suffolk in spring were identified as the least efficient.

The impact on pregnancy success was highly dependent on the embryo transfer strategy. For single embryo transfer, breed, season, and their interaction all had extremely significant effects on pregnancy rate (*p* < 0.001 for all, Table 3). However, for double embryo transfer, none of these factors reached statistical significance. The detailed pregnancy rates across all breed-season combinations are provided in Table S3. Notably, for single embryo transfer, the highest pregnancy rates were achieved by Australian White in winter (83.33%) and autumn (79.40%), and by White-headed Suffolk in autumn (80.36%).

### 3.2. Breed-Specific Impact on Oocyte Yield, Developmental Competence, and Embryo Production Outcomes

A comprehensive comparison of the five sheep breeds revealed significant differences in key parameters of the LOPU-IVEP pipeline (Table 4). East Friesian demonstrated superior oocyte recovery, yielding the highest number of total COCs per ewe (26.15 ± 4.63) and available COCs (18.69 ± 3.04), significantly outperforming Australian White (16.29 ± 1.68 and 10.93 ± 1.38, respectively). East Friesian also yielded the most Grade A oocytes (6.54 ± 2.22).

Black-headed Suffolk most distinct advantage was in developmental competence. It achieved a significantly higher cleavage rate (68.18 ± 10.78%) compared to Black-headed Dorper (43.81 ± 23.83%) and East Friesian (46.32 ± 10.92%), and achieved the highest development rate (37.02 ± 10.39%). Furthermore, oocytes from East Friesian developed into blastocysts at the highest rate (59.38 ± 17.44%), although this was not statistically different from other breeds.

Regarding pregnancy success, East Friesian and Australian White emerged as the most reliable recipients. East Friesian achieved the highest pregnancy rates for both single (73.31 ± 5.82%) and double embryo transfer (75.10 ± 13.14%). Australian White also showed high single-embryo pregnancy rates (70.22 ± 19.40%).

In summary, East Friesian was the most efficient oocyte donor, Black-headed Suffolk oocytes exhibited the highest inherent developmental potential, and East Friesian and Australian White embryos resulted in the most successful pregnancies.

### 3.3. Autumn and Winter Are the Most Favorable Seasons for Embryo Development and Pregnancy Establishment

The analysis of seasonal effects revealed that while COCs quantity remained largely consistent throughout the year, oocyte developmental competence and pregnancy success exhibited significant seasonal variation (Table 5).

Oocyte retrieval efficiency was generally stable across seasons, with no significant differences in the yield of total COCs, available COCs, or Grade A and B COCs. The only exception was the number of Grade C COCs, which was significantly lower in summer (5.70 ± 1.13) compared to spring and winter.

In contrast, a clear pattern emerged for embryonic development. Autumn proved to be the most favorable season, yielding the highest cleavage rate (64.05 ± 16.25%), blastocyst rate (59.02 ± 4.83%), and development rate (37.39 ± 8.67%), with values significantly higher than those in summer. Winter followed as the second most conducive season for embryo production.

This superior developmental competence in the cooler seasons translated directly into higher pregnancy rates. Both autumn and winter achieved the highest success rates for single embryo transfer (73.04 ± 9.70% and 75.31 ± 7.26%, respectively) and double embryo transfer (77.09 ± 12.36% and 74.04 ± 15.37%, respectively). Conversely, summer consistently resulted in the lowest developmental and pregnancy outcomes.

In summary, autumn and winter were identified as the optimal seasons for IVEP, characterized by high oocyte developmental competence and superior pregnancy rates, whereas summer was the least favorable period.

**Figure 4:**
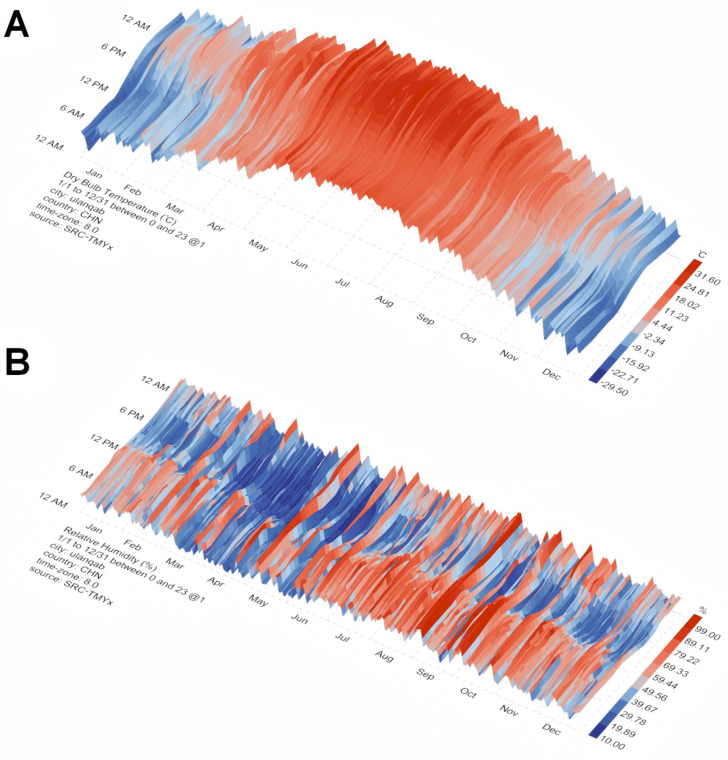
Annual climate profiles of temperature and relative humidity in Ulanqab. (**A**) 3D heat map of monthly average temperature. (**B**) 3D heat map of monthly average relative humidity. Climate data were sourced from the Chinese Standard Weather Data (CSWD). Visualizations were generated using Ladybug Tools v1.6.0.

The seasonal pattern of IVEP efficiency observed in this study is strongly correlated with the local climate of Ulanqab, China (Figure 4 and Figure S1). The superior embryo production and pregnancy outcomes in autumn and winter can be attributed to a combination of moderate temperatures, low humidity, and decreasing photoperiod, which together establish an optimal physiological state for oocyte maturation and embryonic development. In contrast, the heat and humidity of summer consistently suppressed reproductive performance, likely due to heat stress impairing cellular and metabolic processes. Therefore, these findings underscore the necessity of integrating regional climatic data into the planning of annual breeding and embryo transfer schedules to enhance the efficiency of commercial sheep embryo production.

### 3.4. Key LOPU-IVEP Traits Are Not Intercorrelated, Highlighting the Independence of Quantity and Quality Metrics

**Figure 5:**
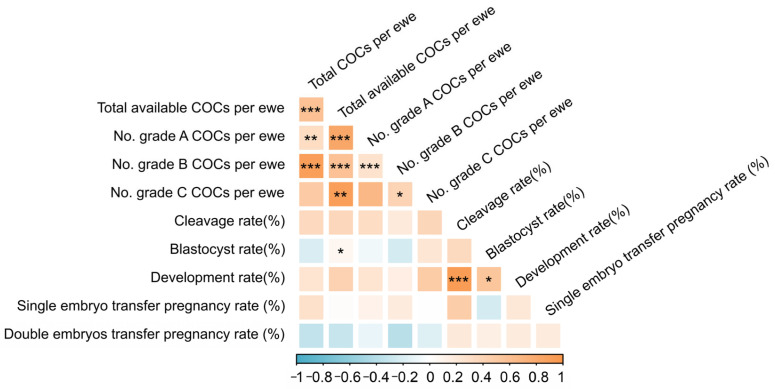
Correlation Heatmap of LOPU-IVEP Efficiency Traits. This heatmap illustrates the correlation coefficients between various traits related to LOPU-IVEP efficiency. The color gradient represents the strength and direction of the Spearman correlation coefficients, with darker shades indicating stronger correlations. Asterisks denote statistical significance at the 0.05 (*), 0.01 (**) and 0.001 (***) levels.

Spearman correlation analysis revealed complex relationships among the key traits in the LOPU-IVEP (Figure 5, Table S4). Strong positive correlations were observed within the oocyte recovery and quality parameters. The number of total COCs per ewe was highly correlated with the number of available COCs (r = 0.746, *p* < 0.001), grade B COCs (r = 0.863, *p* < 0.001), and grade A COCs (r = 0.576, *p* = 0.008). Furthermore, the number of available COCs showed a very strong positive correlation with the number of grade A COCs (r = 0.896, *p* < 0.001).

For developmental competence, a remarkably strong positive correlation was found between the cleavage rate and the subsequent development rate (r = 0.881, *p* < 0.001). The blastocyst rate was also positively correlated with the development rate (r = 0.568, *p* = 0.011) and moderately with the number of available COCs (r = 0.512, *p* = 0.025).

Crucially, no significant correlations were detected between the initial COCs quantity parameters and the key functional quality outcomes. This indicates that the number of oocytes recovered is a poor predictor of their subsequent developmental potential and ultimate success in establishing a pregnancy.

## 4. Discussion

The significantly lower oocyte developmental competence and pregnancy rates observed in summer are strongly correlated with the most challenging climatic conditions in Ulanqab, where July is the hottest and most humid month. This combination of high temperature and humidity induces significant heat stress in sheep, a well-established limiting factor for reproduction [38]. The physiological burden is twofold: Firstly, as metabolic heat production increases, ewes struggle to regulate their core body temperature. Secondly, at the cellular level, heat stress disrupts ovarian function and oocyte maturation by causing abnormal protein folding and the accumulation of damaged cellular components [38]. These insults collectively impair critical organelles such as oocyte mitochondria, which are essential for energy production during embryonic cleavage [39]. This mechanistic cascade directly explains the poor cleavage and blastocyst rates observed in most breeds during summer, a finding consistent with the seasonal reproductive characteristics of sheep. Furthermore, the high rainfall and cloud cover likely contribute to environmental stressors and may indirectly affect animal comfort and feed intake.

In contrast to summer, autumn provides a markedly improved environment for reproduction, characterized by moderate temperatures and the lowest humidity levels of the year, typically ranging between 30% and 50%. It is critical to note that the experimental flocks were housed in modern, standardized sheep housing with proper management. This management system likely played a crucial role in buffering the animals from the extremes of both summer heat and winter cold, thereby allowing the inherent genetic and seasonal potentials to be more accurately expressed. Within this controlled environment, the autumn’s natural climatic conditions facilitate an optimal metabolic and hormonal balance for oocyte development, which is directly evidenced by our data showing the highest cleavage and blastocyst rates.

While winter brings cold conditions, the modern housing effectively mitigated severe cold stress. Furthermore, research suggests that controlled cold exposure may not be entirely detrimental and could even confer benefits [17,40]. Mild cold stress has been shown to stimulate the rapid synthesis of Heat Shock Proteins (HSPs) in ewes [41,42]. These HSPs function as molecular chaperones that help maintain normal protein folding and cellular metabolism within oocytes, thereby enhancing their survival rate and overall stress tolerance. Therefore, the high developmental competence and pregnancy rates observed in winter likely result from a combination of effective environmental buffering by the housing system and the induction of beneficial cellular adaptive mechanisms in the absence of heat stress.

The transition into autumn and winter is marked by decreasing daylight hours, a powerful environmental cue that entrains the natural breeding season in sheep. This photoperiodic signal is primarily mediated by increased melatonin secretion, which in turn regulates the melatonin-follicle-stimulating hormone (FSH) axis [43,44]. The elevated melatonin levels during longer nights are known to promote the activity of follicular granulosa cells and enhance the implantation potential of embryos [45]. Furthermore, the moderate lighting conditions of autumn have been shown to extend the duration of the follicle wave [43]. This neuroendocrine synergy ultimately creates a state of heightened reproductive receptivity. In our system, this physiological state manifests as the observed superior oocyte developmental competence and the highest pregnancy rates following embryo transfers in these seasons.

Beyond seasonal variations, breed (genetic background) emerges as the strongest predictor of oocyte yield and quality. The profound and consistent differences among breeds, such as East Friesian yielding the highest number of total and available COCs while Australian White yielded the lowest, highlight strong genetic determinism for follicular recruitment and oocyte recovery efficiency. This suggests that the choice of donor breed is the primary decision for maximizing raw oocyte yield in a commercial LOPU-IVEP system.

However, a critical divergence was observed between oocyte quantity and developmental competence. Specifically, Black-headed Suffolk oocytes, despite a numerically lower recovery rate than East Friesian, exhibited significantly superior developmental competence, as evidenced by the highest cleavage and development rates. This underscores a fundamental concept: a high oocyte yield does not automatically equate to high embryo production efficiency. The genetic factors controlling the number of ovulatory follicles appear to be distinct from those governing cytoplasmic maturation and epigenetic programming, which are crucial for successful embryonic development.

The significant interaction between breed and season for key developmental parameters (cleavage and development rate) is perhaps the most operationally relevant finding. It demonstrates that the response of a breed to seasonal environmental cues is not uniform. This interaction explains why a single seasonal management policy is suboptimal. The cluster analysis (Figure 3) provides a clear visual representation of this interaction, identifying Black-headed Suffolk in autumn and winter as a superior combination for embryo production. This breed appears to possess a genetic makeup that is particularly responsive to the improving (autumn) or stable (winter) photoperiod and the release from summer heat stress, effectively channeling these favorable conditions into enhanced ooplasmic quality and developmental competence. Conversely, the poor performance of Australian White in summer and Black-headed Dorper in winter highlights breed-specific vulnerabilities. Australian White may be exceptionally susceptible to heat stress, while Black-headed Dorper might be more sensitive to cold stress or the metabolic demands of thermoregulation, thereby diverting energy away from reproductive processes.

Our data further reveal that single-embryo transfer pregnancy rates are significantly influenced by both breed and season (*p* < 0.001), whereas double-embryo transfer demonstrates relative stability across these variables. This key finding indicates that double-embryo transfer can serve as an effective buffering strategy against challenges posed by unfavorable breed characteristics or seasonal stress, potentially through mechanisms such as embryo cooperativity that enhance implantation reliability [46,47]. Conversely, this finding also underscores that single-embryo transfer, while susceptible to physiological and environmental variations, remains the optimal strategy for maximizing the utilization of precious embryos—particularly those that are sexed or genetically modified—by completely avoiding the maternal and neonatal risks associated with multiple pregnancies, such as abortion, dystocia, and low birth weight [47,48]. Therefore, single-embryo transfer is the preferred approach in advanced breeding programs aimed at high-value, controlled genetic output, such as nucleus herd improvement and transgenic animal production. Future work will track lambing outcomes to comprehensively evaluate efficiency from conception to parturition, further informing strategic embryo transfer decisions based on embryo value, recipient suitability, and production objectives.

Based on our integrative analysis of the breed and season effects, we propose a precision strategy for commercial sheep embryo production to maximize annual efficiency and economic return. Align the LOPU and ET schedule for each breed with its identified optimal season. The production cycle for Black-headed Suffolk, the breed with the highest oocyte developmental competence, should be concentrated in autumn and winter to capitalize on this synergistic advantage. Reduce LOPU frequency for Black-headed Suffolk and Black-headed Dorper during summer to avoid the collection of low-competence oocytes. Concurrently, implement enhanced feeding management and cooling strategies to alleviate heat stress during this period. East Friesian, as the highest oocyte yielder, should be utilized as a donor primarily during its most favorable seasons to maximize raw material output for embryo production.

In conclusion, we recommend that autumn be prioritized as the prime window for oocyte collection and embryo transfer. We further advise that the majority of the annual embryo production quota be concentrated in the autumn and winter seasons. By adopting this breed-season integrated management approach, commercial operations can strategically leverage genetic strengths and environmental synergies to achieve superior productivity.

## 5. Conclusions

The efficiency of large-scale commercial in vitro sheep embryo production is determined not in isolation but by the significant interaction between genetic background (breed) and environmental conditions (season). Autumn and Winter constitute the optimal window for embryo production, characterized by climatic conditions that promote superior oocyte developmental competence, leading to the highest pregnancy rates. The absence of correlation between oocyte quantity and functional quality (development and pregnancy) underscores the necessity for a dual selection criterion in breeding programs, prioritizing both high yield and high developmental potential. In summary, this study provides a robust scientific basis for implementing a precision, breed-season integrated management strategy. By scheduling LOPU operations to leverage the synergistic effects of specific breed-season combinations, commercial sheep embryo production can achieve significant gains in both productivity and economic returns.

## Figures and Tables

**Table 1:** Effects of breed and season on laparoscopic ovum pick-up (LOPU) oocyte retrieval efficiency and quality in sheep.

	Breed		Season		Breed × Season	
	*X* ^2^	*p*	*X* ^2^	*p*	*X* ^2^	*p*
No. total COCs per ewe	37.125	<0.001	1.759	0.624	7.347	0.770
No. available COCs per ewe	29.073	<0.001	6.409	0.093	7.991	0.714
No. grade A COCs per ewe	33.110	<0.001	1.477	0.688	7.205	0.782
No. grade B COCs per ewe	9.997	0.04	2.561	0.464	3.205	0.988
No. grade C COCs per ewe	2.747	0.601	4.778	0.189	2.229	0.998

Data were analyzed using GLM. The chi-square (*X*^2^) statistics are Wald tests for the fixed effects. COCs, cumulus-oocyte complexes.

**Table 2:** Effects of breed and season on in vitro embryonic development in sheep.

	Breed		Season		Breed × Season	
	F	*p*	F	*p*	F	*p*
Cleavage rate (%)	7.905	<0.001	10.722	<0.001	4.262	0.001
Blastocyst rate (%)	1.198	0.331	3.435	0.028	0.500	0.889
Development rate (%)	2.909	0.037	9.196	<0.001	2.363	0.029

Data were analyzed using two-way ANOVA following arcsine square-root transformation of percentage data. The F-values and *p*-values are presented for the main effects of breed and season and their interaction. Development rate was calculated as the proportion of cleaved oocytes that developed to the blastocyst stage.

**Table 3:** Effects of breed and season on pregnancy rate following single or double embryo transfer in sheep.

	Breed		Season		Breed × Season	
	*X* ^2^	*p*	*X* ^2^	*p*	*X* ^2^	*p*
Single embryo transfer pregnancy rate (%)	38.803	<0.001	36.525	<0.001	107.473	<0.001
Double embryos transfer pregnancy rate (%)	2.259	0.307	9.253	0.099	8.284	0.111

Data were analyzed using binary logistic regression. The chi-square (*X*^2^) statistics are Wald tests for the fixed effects.

**Table 4:** Comparative analysis of oocyte retrieval efficiency, developmental competence, and pregnancy outcomes across five sheep breeds.

	Black-Headed Suffolk	White-Headed Suffolk	Australian White	Black-Headed Dorper	East Friesian
No. of donor ewes	3204	699	760	1109	568
Total COCs per ewe	19.79 ± 2.22 ^bc^	17.57 ± 2.98 ^cd^	16.29 ± 1.68 ^d^	23.08 ± 3.28 ^ab^	26.15 ± 4.63 ^a^
Total available COCs per ewe	15.57 ± 2.50 ^ab^	12.93 ± 2.40 ^bc^	10.93 ± 1.38 ^c^	13.38 ± 4.98 ^bc^	18.69 ± 3.04 ^a^
No. grade A COCs	4.43 ± 1.55 ^b^	3.14 ± 1.10 ^b^	2.79 ± 0.70 ^c^	2.77 ± 2.39 ^b^	6.54 ± 2.22 ^a^
No. grade B COCs	3.64 ± 0.84 ^bc^	3.14 ± 0.66 ^bc^	2.50 ± 0.52 ^c^	3.92 ± 2.02 ^ab^	4.92 ± 1.12 ^a^
No. grade C COCs	7.50 ± 0.85 ^a^	6.64 ± 1.34 ^ab^	5.64 ± 1.01 ^b^	6.69 ± 1.60 ^ab^	7.23 ± 1.36 ^a^
Cleavage rate (%)	68.18 ± 10.78 ^a^	47.28 ± 16.27 ^ab^	54.38 ± 23.41 ^ab^	43.81 ± 23.83 ^b^	46.32 ± 10.92 ^b^
Blastocyst rate (%)	53.44 ± 8.50 ^a^	55.54 ± 12.01 ^a^	47.79 ± 13.68 ^a^	46.11 ± 18.66 ^a^	59.38 ± 17.44 ^a^
Development rate (%)	37.02 ± 10.39 ^a^	26.88 ± 10.52 ^a^	28.08 ± 17.05 ^a^	21.58 ± 16.17 ^a^	28.83 ± 10.43 ^a^
Single embryo transfer pregnancy rate (%)	62.05 ± 12.08 ^a^	57.89 ^a^	70.22 ± 19.40 ^a^	67.11 ± 11.02 ^a^	73.31 ± 5.82 ^a^
Double embryos transfer pregnancy rate (%)	68.61 ± 4.65 ^a^	-	67.59 ± 32.15 ^a^	64.04 ± 3.35 ^a^	75.10 ± 13.14 ^a^

Data are presented as mean ± SD. a, b, c, d Values with different superscripts within a row differ significantly (*p* < 0.05). Values with similar superscripts within rows mean no statistically differences (*p* > 0.05). “-” indicates that no data were recorded. Values without ± SD denote no replicate records.

**Table 5:** Comparative analysis of oocyte retrieval efficiency, developmental competence, and pregnancy outcomes across seasons.

	Spring	Summer	Autumn	Winter
No. of donor ewes	1830	1615	1403	1492
Total COCs per ewe	21.81 ± 4.58 ^a^	18.85 ± 3.63 ^a^	19.60 ± 5.06 ^a^	21.18 ± 5.45 ^a^
Total available COCs per ewe	15.10 ± 4.04 ^a^	12.55 ± 3.10 ^a^	13.30 ± 2.21 ^a^	15.76 ± 4.91 ^a^
No. grade A COCs	4.10 ± 2.32 ^a^	3.75 ± 1.86 ^a^	3.30 ± 0.95 ^a^	4.24 ± 2.84 ^a^
No. grade B COCs	4.14 ± 1.59 ^a^	3.10 ± 0.91 ^a^	3.10 ± 0.74 ^a^	3.82 ± 1.59 ^a^
No. grade C COCs	6.86 ± 1.06 ^a^	5.70 ± 1.13 ^b^	6.90 ± 1.20 ^ab^	7.71 ± 1.36 ^a^
Cleavage rate (%)	50.86 ± 14.43 ^ab^	50.86 ± 14.43 ^b^	64.05 ± 16.25 ^a^	58.63 ± 15.51 ^a^
Blastocyst rate (%)	52.66 ± 12.47 ^ab^	52.66 ± 12.47 ^b^	59.02 ± 4.83 ^a^	57.54 ± 8.71 ^a^
Development rate (%)	27.67 ± 11.62 ^ab^	27.67 ± 11.62 ^b^	37.39 ± 8.67 ^a^	33.70 ± 10.26 ^a^
Single embryo transfer pregnancy rate (%)	63.00 ± 13.76 ^a^	58.04 ± 9.25 ^a^	73.04 ± 9.70 ^a^	75.31 ± 7.26 ^a^
Double embryos transfer pregnancy rate (%)	70.10 ± 8.19 ^a^	54.96 ± 20.84 ^a^	77.09 ± 12.36 ^a^	74.04 ± 15.37 ^a^

Data are presented as mean ± SD. a, b Values with different superscripts within a row differ significantly (*p* < 0.05). Values with similar superscripts within rows mean no statistically differences (*p* > 0.05).

## Data Availability

The data are available from the first author, Yubing Wang (wangyubing911@163.com), upon request.

## Supplementary Materials

The following supporting information can be downloaded at: https://www.mdpi.com/article/10.3390/ani15223354/s1, Figure S1: Annual profiles of solar radiation and precipitation in Ulanqab. Table S1: Statistical data of COCs from donor ewes across different breeds and seasons. Table S2: Statistics on cleavage rate, blastocyst rate, and development rate across different breeds and seasons. Table S3: Pregnancy rate following single or double embryo transfer across different breeds and seasons. Table S4: Correlations among the traits.
