# Supplementary material for: Breed and Season: Key Determinants of Efficiency in Large-Scale Commercial In Vitro Sheep Embryo Production
Source: Animals (Basel). 2025 Nov 20;15(22):3354. doi: 10.3390/ani15223354 (PMC12649618; doi:10.3390/ani15223354)
Supplement: Supplementary file 1 [file animals-15-03354-s001.zip › Figure S1.pdf]

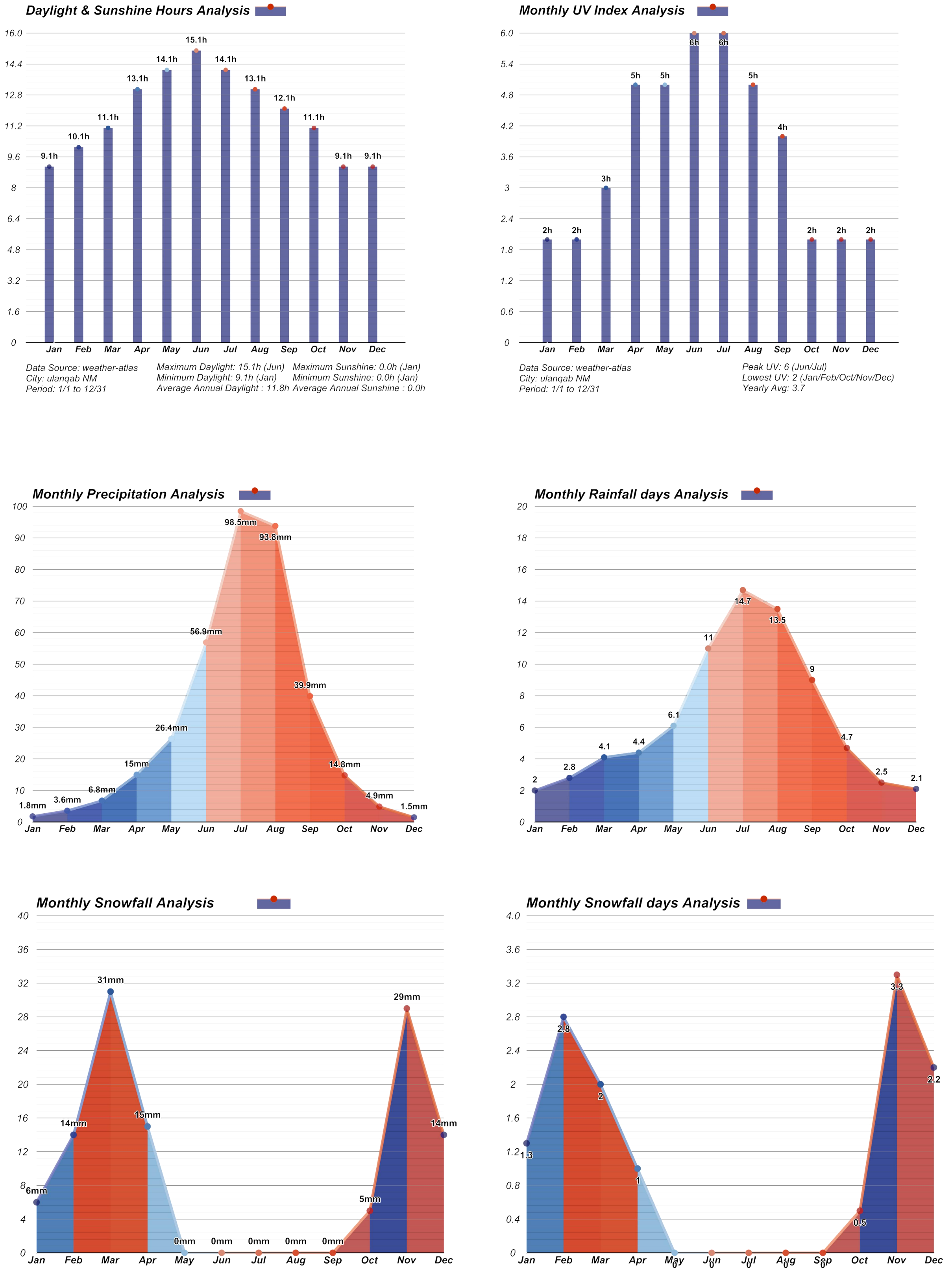

Figure S1. Annual profiles of solar radiation and precipitation in Ulanqab.
Comprehensive climate data illustrating monthly daylength and ultraviolet (UV) index, and monthly rainfall/snowfall amounts and the number of precipitation days. Climate data were sourced from the Chinese Standard Weather Data (CSWD). Visualizations were generated using Ladybug Tools v1.6.0.
